# Structured exercise, cognitive–social engagement, and cognitive impairment‐free life expectancy across four longitudinal aging cohorts

**DOI:** 10.1002/alz.71854

**Published:** 2026-10-13

**Authors:** Xiangwei Li, Ying Liu, Junyu Sun, Hui Zhang, Yaqian Xu, Chongyu Ding, Yanting Zhang, Tianlang Tong, Zhaojun Wang, Wei Lyu, Xian Cui

**Affiliations:** ^1^ School of Global Health, Chinese Centre for Tropical Diseases Research Shanghai Jiao Tong University School of Medicine Shanghai China; ^2^ Hainan International Medical Center Shanghai Jiao Tong University School of Medicine Hainan China; ^3^ China Academy of Chinese Medical Sciences Beijing China; ^4^ Institute of Psychiatry Psychology & Neuroscience King's College London London UK; ^5^ Department of Infectious Diseases Peking Union Medical College Hospital Chinese Academy of Medical Sciences & Peking Union Medical College Beijing China; ^6^ Diagnostic Imaging Center Shanghai Children's Medical Center Shanghai Jiao Tong University School of Medicine Shanghai China

**Keywords:** aging cohorts, cognitive aging, cognitive impairment, cognitive–social engagement, life expectancy, multistate life table, structured exercise

## Abstract

**INTRODUCTION:**

Whether cognitive–social engagement can compensate for insufficient structured exercise in cognitive aging remains unclear.

**METHODS:**

We harmonized repeated data from four aging cohorts across Asia, North America, and Europe. Structured exercise and cognitive–social engagement were cross‐classified at each wave into low–low (LL), high exercise–low engagement (HL), low exercise–high engagement (LH), and high–high (HH). Cohort‐specific Cox models and multistate life tables estimated incident cognitive impairment and cognitive impairment‐free life expectancy.

**RESULTS:**

Cognitive impairment‐free life expectancy from age 50 was 17.5, 23.2, 20.1, and 24.7 years for the low exercise + low cognitive‐social engagement (LL), high exercise + low cognitive‐social engagement (HL), low exercise + high cognitive‐social engagement (LH), and high exercise + high cognitive‐social engagement (HH) groups, respectively.

**DISCUSSION:**

Structured exercise and cognitive–social engagement showed combined, non‐interchangeable benefits for cognitive aging. These findings support integrated lifestyle strategies for dementia prevention across diverse aging populations, health systems, and prevention settings worldwide.

## Highlights

- Structured exercise and cognitive–social engagement were jointly linked to cognitive aging.
- Four aging cohorts were harmonized using time‐updated exposure profiles.
- The high–high profile showed the lowest incident cognitive impairment risk.
- High–high was associated with 7.2 additional cognitive impairment–free years.
- Findings support integrated lifestyle strategies for dementia prevention.

## 1 BACKGROUND

Dementia and cognitive impairment are growing public health challenges in aging societies. Global dementia cases are projected to rise from 57 million in 2019 to ≈ 153 million by 2050.^1^ As disease‐modifying treatments remain limited at the population level, prevention has increasingly focused on modifiable lifestyle factors. The 2024 Lancet Commission estimated that ≈ 45% of dementia cases may be preventable or delayable, highlighting physical inactivity and social isolation as key targets.^2^

Physical activity is one of the most consistently studied behavioral factors in cognitive aging.^3^, ^4^ Higher physical activity has been associated with lower dementia risk, although associations vary by activity domain, intensity, and timing.^5^ Importantly, structured or leisure‐time exercise may differ from general daily movement because it is purposeful and may provide stronger cardiorespiratory, neuromuscular, and metabolic stimulation.^3^, ^6^ However, many previous studies have treated physical activity as a broad exposure, leaving uncertainty about the specific contribution of structured exercise to cognitive aging.

Cognitive and social engagement may also protect cognitive health by supporting cognitive reserve, social connectedness, emotional regulation, and maintenance of daily routines. Classic prospective evidence showed that cognitively stimulating leisure activities were associated with lower dementia risk.^7^ More recent studies have further linked cognitive and social leisure activities with later‐life cognitive and brain health,^8^ while meta‐analytic evidence suggests that poor social relationships are associated with cognitive decline.^9^ Nevertheless, most studies have examined physical activity, cognitive activity, or social engagement separately. Although cognitive engagement and social engagement are conceptually distinct and may influence cognitive aging through different pathways,^10^ ^11^ they frequently overlap in later life activities, such as volunteering and community participation.^12^ Therefore, we considered cognitive–social engagement as a broader behavioral domain to facilitate harmonized analyses across cohorts while acknowledging the potentially distinct contributions of cognitive and social engagement.

A key unresolved question is whether cognitive–social engagement can compensate for insufficient structured exercise, or whether maintaining both behaviors provides the greatest cognitive benefit. Although physical activity and social engagement have been examined jointly in some longitudinal studies, prior work has mainly focused on cognitive‐score trajectories or specific subgroups rather than direct substitutability between lifestyle domains.^13^ Existing studies have also often relied on baseline exposure assessment and relative risk estimates, whereas both exercise and engagement behaviors change over time.^14^, ^15^ Moreover, translating associations into cognitive impairment–free life expectancy may provide a more interpretable measure of public health impact than hazard ratios alone.^16^

To address these gaps, we harmonized repeated longitudinal data from four aging cohorts: the China Health and Retirement Longitudinal Study (CHARLS), Health and Retirement Study (HRS), English Longitudinal Study of Ageing (ELSA), and Korean Longitudinal Study of Ageing (KLoSA). We jointly classified structured exercise and cognitive–social engagement into time‐updated dual‐exposure profiles and examined incident cognitive impairment. We further assessed pairwise comparisons, long‐term exposure patterns, and cognitive impairment–free life expectancy from age 50 using multistate life table methods. We hypothesized that maintaining both structured exercise and cognitive–social engagement would be associated with the lowest risk of cognitive impairment and the longest cognitive impairment–free life expectancy.

**RESEARCH IN CONTEXT**

1. **Systematic review**: We reviewed prior epidemiological, clinical, and mechanistic studies on physical activity, cognitive activity, social engagement, dementia risk, and cognitive impairment–free or dementia‐free life expectancy. Previous studies have generally shown that higher physical activity, cognitively stimulating activities, and social engagement are associated with better cognitive outcomes and lower dementia risk. However, most studies examined these lifestyle domains separately, relied on baseline exposure assessment, or focused on cognitive‐score trajectories rather than incident cognitive impairment. Few studies have directly tested whether cognitive–social engagement can compensate for insufficient structured exercise, and fewer have translated such associations into cognitive impairment‐free life expectancy.
2. **Interpretation**: Using repeated data from four longitudinal aging cohorts across Asia, North America, and Europe, we found that structured exercise and cognitive–social engagement were jointly associated with incident cognitive impairment. Compared to low exercise + low cognitive–social engagement, all favorable profiles were associated with lower risk, with the strongest association observed for high exercise + high cognitive–social engagement. Direct comparisons showed that the high–high profile was associated with lower risk than either single‐high profile, whereas high exercise alone and high cognitive–social engagement alone did not clearly differ. From age 50, the high–high profile was associated with 24.7 cognitive impairment–free years, compared to 17.5 years in the low–low profile, corresponding to 7.2 additional years lived free of cognitive impairment.
3. **Future directions**: Future studies should validate these findings using more detailed and harmonized measures of exercise intensity, cognitive activity, and social participation, ideally incorporating device‐based activity assessment, neuropsychological batteries, biomarkers, and neuroimaging. Further work is needed to clarify whether the observed combined benefit reflects additive or synergistic effects and to identify the biological and psychosocial mechanisms linking structured exercise and cognitive–social engagement to cognitive aging. Intervention studies should evaluate whether integrated programs combining structured exercise with cognitively stimulating and socially meaningful activities can delay cognitive impairment and extend cognitive impairment–free life expectancy.

## 2 METHODS

### 2.1 Study design and data sources

This study was designed as a harmonized longitudinal multi‐cohort analysis of structured exercise, cognitive–social engagement, and cognitive aging among middle‐aged and older adults. We used repeated measures from four population‐based aging cohorts: CHARLS,^17^ HRS,^18^ ELSA,^19^ and KLoSA.^20^ CHARLS, HRS, ELSA, and KLoSA are longitudinal aging surveys from China, the United States, England, and Korea, respectively. They were designed to collect repeated information on health, socioeconomic status, family and social circumstances, cognitive function, physical function, chronic diseases, and mortality among middle‐aged and older adults, thereby providing comparable data resources for cross‐national aging research.

Participants were eligible if they were aged ≥ 50 years at baseline, had information on structured exercise and cognitive–social engagement, and had at least one follow‐up assessment for cognitive status. For the incident cognitive impairment analysis, participants with cognitive impairment at baseline were excluded. Participants with fewer than two eligible survey waves or missing baseline dual‐exposure classification were also excluded. The final analytic sample and cohort‐specific exclusions are shown in Figure 1. Variables were harmonized according to a prespecified cohort‐mapping protocol before analysis. Each cohort was analyzed separately using the same analytic framework, and cohort‐specific estimates were subsequently synthesized.

**FIGURE 1:**
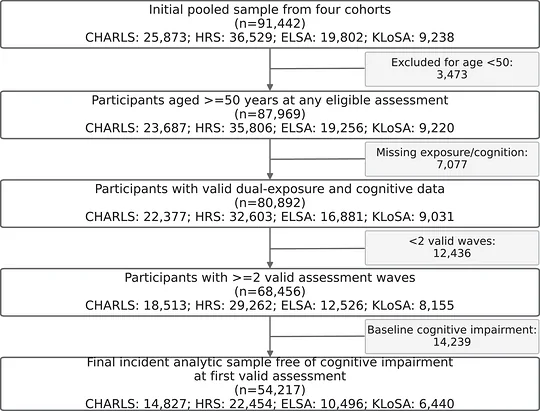
Flowchart of participant selection for the exercise‐engagement cognitive aging analysis. The initial pooled sample included participants from four longitudinal aging cohorts: CHARLS, HRS, ELSA, and KLoSA. Participants were excluded if they were younger than 50 years at any eligible assessment, lacked valid dual‐exposure or cognitive data, had fewer than two valid assessment waves, or had cognitive impairment at the first valid assessment. The final incident analytic sample included participants free of cognitive impairment at baseline. CHARLS, China Health and Retirement Longitudinal Study; ELSA, English Longitudinal Study of Ageing; HRS, Health and Retirement Study; KLoSA, Korean Longitudinal Study of Ageing.

### 2.2 Assessment of structured exercise and cognitive–social engagement

The primary exposures were structured exercise and cognitive–social engagement. Structured exercise was defined as purposeful, planned, or leisure‐time physical activity performed for fitness, recreation, or health. Because questionnaire wording differed across cohorts, cohort‐specific items capturing regular exercise, vigorous or moderate physical activity, sports, or leisure‐time physical activity were mapped onto a common binary indicator of low versus high structured exercise. Harmonization was performed at the construct level rather than by imposing identical absolute thresholds across cohorts. Exposure categories were then defined using cohort‐specific response options and measurement characteristics. Therefore, high and low categories represent relative differences within each cohort and should not be interpreted as universal physical activity guideline thresholds. The detailed cohort‐specific operational definitions are provided in Table S1 in supporting information.

Cognitive–social engagement was defined as participation in cognitively stimulating or socially interactive activities, including social contact, participation in clubs or community groups, hobbies, volunteering, religious or cultural activities, games, educational activities, internet use, or other cohort‐available engagement domains. Although cognitive engagement and social engagement are conceptually distinct, they were combined into a harmonized cognitive–social engagement domain because these domains were not consistently assessed separately across cohorts. Cohort‐specific engagement items were first recoded to indicate participation frequency or presence of engagement and then harmonized into low versus high cognitive–social engagement using cohort‐specific thresholds. This approach preserved within‐cohort comparability while allowing for cultural and survey‐design differences in how engagement was measured.

At each wave, structured exercise and cognitive–social engagement were cross‐classified into four mutually exclusive dual‐exposure categories: low exercise + low cognitive–social engagement (LL), high exercise + low cognitive–social engagement (HL), low exercise + high cognitive–social engagement (LH), and high exercise + high cognitive–social engagement (HH). LL was considered the least favorable profile and served as the reference category in the main analyses unless otherwise specified. For primary analyses, the dual‐exposure category was treated as a time‐updated exposure, with exposure status updated at each available survey wave. The detailed operational definition of wave‐specific dual‐exposure categories are provided in Table S2 in supporting information.

For long‐term pattern analyses, repeated exposure information across the first five observed eligible waves was summarized into predominant dual‐exposure pattern groups: persistently low–low, persistently high–high, stable single‐high, improving, deteriorating, and fluctuating. Pattern groups were assigned hierarchically according to prespecified definitions. Participants classified as persistently low–low or persistently high–high had at least four of the first five eligible waves in the corresponding dual‐exposure category. Stable single‐high indicated repeated classification in either HL or LH. Improving and deteriorating patterns were defined by sustained movement toward or away from more favorable dual‐exposure levels across the first five waves, and all remaining participants were classified as fluctuating. The detailed operational definition of predominant five‐wave dual‐exposure pattern groups are provided in Table S3 in supporting information.

### 2.3 Ascertainment of incident cognitive impairment

The primary outcome was incident cognitive impairment. Cognitive status was assessed repeatedly in each cohort using cohort‐available cognitive tests and derived cognitive status indicators. Cognitive domains included episodic memory, orientation, attention or numeracy, and executive‐related tasks, depending on the cohort. In HRS, cognitive status was informed by the Langa–Weir classification, which maps cognitive test and proxy interview information into normal cognition, cognitive impairment no dementia, and dementia categories.^21^ In the other cohorts, cognitive impairment was harmonized using cohort‐specific cognitive status variables or cognitive test–based classifications that distinguished normal cognition from cognitive impairment. All cohort‐specific cognitive definitions were mapped onto a common binary outcome: no cognitive impairment versus cognitive impairment.

Incident cognitive impairment was defined as the first follow‐up wave at which a participant was classified as cognitively impaired among those free of cognitive impairment at baseline. Participants were followed from baseline until incident cognitive impairment, death, loss to follow‐up, or the end of the available follow‐up period, whichever occurred first. For analyses of life expectancy with and without cognitive impairment, death was incorporated as an absorbing state in the multistate life table framework.

### 2.4 Covariates

Covariates were selected a priori based on previous literature and their potential role as confounders of the association between lifestyle behaviors and cognitive aging. Harmonized covariates included age, sex, education, marital or partner status, smoking status, alcohol consumption, body mass index, and chronic conditions. Chronic conditions included hypertension, diabetes, heart disease, stroke, and other cohort‐available physician‐diagnosed conditions where available. Depressive symptoms, self‐rated health, and functional limitation were additionally considered in sensitivity analyses, because these factors may be confounders but may also partially reflect early cognitive or functional decline. To reduce overadjustment, variables likely to lie on the pathway from lifestyle behavior to cognitive impairment were not included in the primary model unless prespecified as sensitivity covariates.

### 2.5 Statistical analysis

All analyses were conducted separately within each cohort using a harmonized analytic protocol. Baseline characteristics were summarized across the four baseline dual‐exposure groups using means and standard deviations for continuous variables and counts and percentages for categorical variables. Differences in distributions across cohorts and exposure groups were described rather than interpreted as formal hypothesis tests.

Associations between time‐updated dual‐exposure categories and incident cognitive impairment were estimated using Cox proportional hazards models, with hazard ratios (HRs) and 95% confidence intervals (CIs) calculated within each cohort. The primary time scale was follow‐up time, with age included as a covariate; sensitivity analyses considered alternative time‐scale specifications where appropriate. The proportional hazards assumption was assessed using Schoenfeld residuals and graphical diagnostics.

Three nested models were fitted. Model 1 adjusted for age and sex. Model 2 additionally adjusted for sociodemographic characteristics, including education and marital or partner status. Income and wealth measures were not included because they were not consistently available or harmonized across all cohorts. Model 3 further adjusted for lifestyle factors, body mass index, and baseline chronic conditions. The fully adjusted model was used as the primary model. LL served as the reference category in the main analyses.

To directly evaluate whether cognitive–social engagement could compensate for insufficient structured exercise, we prespecified pairwise comparisons between active dual‐exposure profiles: HL versus LH, HH versus HL, and HH versus LH. These comparisons were estimated by changing the reference category within the same fully adjusted model. To assess whether structured exercise and cognitive–social engagement showed synergistic or additive benefits, we also fitted models including the main effects of structured exercise and cognitive–social engagement together with their multiplicative interaction term. Formal interaction tests were performed within each cohort and then summarized across cohorts. If the interaction term was not statistically significant, the combined HH profile was interpreted as reflecting combined or additive benefit rather than statistical synergy.

Cohort‐specific estimates were pooled using random‐effects meta‐analysis to account for between‐cohort heterogeneity. Between‐cohort heterogeneity was quantified using the *I* ^2^ statistic and Cochran *Q* test. Forest plots were used to display both cohort‐specific and pooled estimates. This cohort‐specific‐then‐pooled strategy was chosen to preserve cohort‐specific coding and sampling structures while providing an overall summary estimate.^22^

To examine whether sustained or changing lifestyle profiles carried prognostic information beyond single‐wave exposure, we conducted long‐term pattern analyses among participants with repeated exposure information across the first five eligible waves. Predominant pattern groups were related to subsequent incident cognitive impairment using Cox models among participants who remained free of cognitive impairment through the pattern‐definition period. Persistently low–low served as the reference group. Models used the same covariate adjustment structure as the primary analysis, with covariates assessed at or before the beginning of outcome follow‐up when possible.

We further visualized transitions across the first five waves using alluvial plots. These plots described changes in the joint distribution of structured exercise and cognitive–social engagement over time but were not used for formal inference.

To translate relative associations into absolute measures of cognitive health, we estimated cognitive impairment–free life expectancy from age 50. We used a three‐state multistate life table framework with the following states: alive without cognitive impairment, alive with cognitive impairment, and death. Transitions were allowed from alive without cognitive impairment to cognitive impairment, from alive without cognitive impairment to death, and from cognitive impairment to death. Death was treated as an absorbing state. This approach follows prior studies that estimated life expectancy with and without dementia or Alzheimer's disease (AD) dementia using multistate life table methods.^16^, ^23^

Age‐specific transition rates were estimated for each dual‐exposure group and used to derive expected years lived without cognitive impairment, years lived with cognitive impairment, and total remaining life expectancy from age 50. We also calculated absolute gains in cognitive impairment–free years for key comparisons: HL versus LH, HH versus HL, HH versus LH, and HH versus LL. In addition, we estimated the proportion of remaining life lived free of cognitive impairment, calculated as cognitive impairment–free life expectancy divided by total remaining life expectancy.

Several sensitivity analyses were performed. First, to reduce potential reverse causation, we repeated analyses after excluding cognitive impairment events occurring within the first 1 and 2 years of follow‐up. Second, we repeated analyses using alternative exposure cut‐points for structured exercise and cognitive–social engagement. Third, we fitted competing‐risk models treating death before cognitive impairment as a competing event using Fine–Gray subdistribution hazard models.^24^ Fourth, we additionally adjusted for depressive symptoms, self‐rated health, functional limitation, and baseline chronic disease burden where available. Fifth, we performed multiple imputation by chained equations for missing covariate data under the missing‐at‐random assumption, incorporating exposure, outcome, follow‐up time, and all covariates included in the analytic models; estimates were combined using Rubin rules.^25^ Sixth, we repeated the meta‐analysis after omitting one cohort at a time to evaluate whether any single cohort disproportionately influenced the pooled estimates. Finally, E‐values were calculated for key pooled associations to assess the minimum strength of association that an unmeasured confounder would need to have with both exposure and outcome to fully explain the observed association.^26^

Prespecified subgroup analyses were conducted according to age group, sex, education, body mass index category, and baseline multimorbidity where available. Subgroup‐specific HRs were estimated using the fully adjusted model, and interaction *P* values were obtained by including cross‐product terms between the dual‐exposure comparison and subgroup variable. Subgroup analyses were interpreted as exploratory assessments of heterogeneity rather than as independent confirmatory analyses.

All statistical tests were two sided, and *P* values < 0.05 were considered statistically significant. Analyses were conducted using R or Python according to the harmonized analytic protocol.

## 3 RESULTS

### 3.1 Study population and baseline characteristics

The participant flow is shown in Figure 1. Among 91,442 participants in the initial pooled sample from four cohorts, 87,969 were aged ≥ 50 years at an eligible assessment, 80,892 had valid dual‐exposure and cognitive data, and 68,456 had at least two valid assessment waves. After excluding participants with cognitive impairment at the first valid assessment, 54,217 participants were included in the incident cognitive impairment analysis.

Baseline characteristics are summarized in Table 1. The baseline analytic dataset included 47,254 participants: 14,679 from CHARLS, 18,279 from HRS, 7995 from ELSA, and 6301 from KLoSA. The pooled mean age was 59.2 years, and 53.8% were women. At baseline, 26.8% were classified as low exercise + low cognitive–social engagement, 13.2% as high exercise + low cognitive–social engagement, 35.2% as low exercise + high cognitive–social engagement, and 24.8% as high exercise + high cognitive–social engagement. Cohort‐specific exposure definitions and pattern definitions are shown in Tables S1–S3.

**TABLE 1:** Baseline characteristics of participants included in the four‐cohort cognitive–social analysis.

Characteristic	CHARLS	HRS	ELSA	KLOSA	Pooled
Total	14,827	22,454	10,496	6440	54,217
Age, mean (SD)	59.30 (8.63)	61.16 (9.61)	61.68 (9.20)	63.73 (9.54)	61.06 (9.36)
Female, *n* (%)	6,974 (47.0)	13,010 (57.9)	5,708 (54.4)	3542 (55.0)	29,234 (53.9)
Low education, *n* (%)	12,498 (84.3)	2978 (13.3)	3150 (32.7)	3749 (58.2)	22,375 (41.9)
Middle education, *n* (%)	1927 (13.0)	13,643 (60.8)	4742 (49.2)	2025 (31.4)	22,337 (41.9)
High education, *n* (%)	402 (2.7)	5830 (26.0)	1743 (18.1)	666 (10.3)	8641 (16.2)
Married/partnered, *n* (%)	13,197 (89.0)	14,343 (63.9)	7489 (71.4)	5231 (81.2)	40,260 (74.3)
BMI, mean (SD)	23.75 (3.89)	28.54 (6.01)	28.07 (5.09)	23.36 (2.72)	26.63 (5.56)
Current smoking, *n* (%)	4746 (32.0)	3927 (17.6)	1717 (16.4)	1151 (17.9)	11,541 (21.3)
Current drinking, *n* (%)	5476 (36.9)	13,781 (61.4)	9525 (90.8)	2427 (37.7)	31,209 (57.6)
Hypertension, *n* (%)	4065 (27.5)	10,492 (46.7)	3405 (32.4)	2306 (35.8)	20,268 (37.4)
Diabetes, *n* (%)	1139 (7.7)	3539 (15.8)	649 (6.2)	957 (14.9)	6284 (11.6)
Heart disease, *n* (%)	1946 (13.1)	3524 (15.7)	1385 (13.2)	427 (6.6)	7282 (13.4)
Stroke, *n* (%)	432 (2.9)	992 (4.4)	242 (2.3)	241 (3.7)	1907 (3.5)
Low exercise + low CSE, *n* (%)	4879 (32.9)	4736 (21.1)	1913 (18.2)	2952 (45.8)	14,480 (26.7)
High exercise + low CSE, *n* (%)	2396 (16.2)	3077 (13.7)	435 (4.1)	1509 (23.4)	7417 (13.7)
Low exercise + high CSE, *n* (%)	5315 (35.8)	7620 (33.9)	5167 (49.2)	1113 (17.3)	19,215 (35.4)
High exercise + high CSE, *n* (%)	2237 (15.1)	7021 (31.3)	2981 (28.4)	866 (13.4)	13,105 (24.2)

*Note*: Values are mean (SD) or *n* (%). The analytic sample included participants aged ≥ 50 years ∖, with at least two eligible assessment waves, non‐missing baseline dual‐exposure classification, and no cognitive impairment at baseline.

Abbreviations: BMI, body mass index; CHARLS, China Health and Retirement Longitudinal Study; CSE, cognitive–social engagement; ELSA, English Longitudinal Study of Ageing; HRS, Health and Retirement Study; KLoSA, Korean Longitudinal Study of Ageing; SD, standard deviation.

### 3.2 Main associations with incident cognitive impairment

Cohort‐specific and pooled associations are shown in Figure 2. Compared to low exercise + low cognitive–social engagement, all favorable dual‐exposure profiles were associated with lower hazards of incident cognitive impairment. The pooled HR was 0.80 for high exercise + low cognitive–social engagement (95% CI, 0.73–0.88), 0.79 for low exercise + high cognitive–social engagement (95% CI, 0.66–0.95), and 0.70 for high exercise + high cognitive‐social engagement (95% CI, 0.61–0.81). The high–high profile showed the largest risk reduction, although between‐cohort heterogeneity was substantial.

**FIGURE 2:**
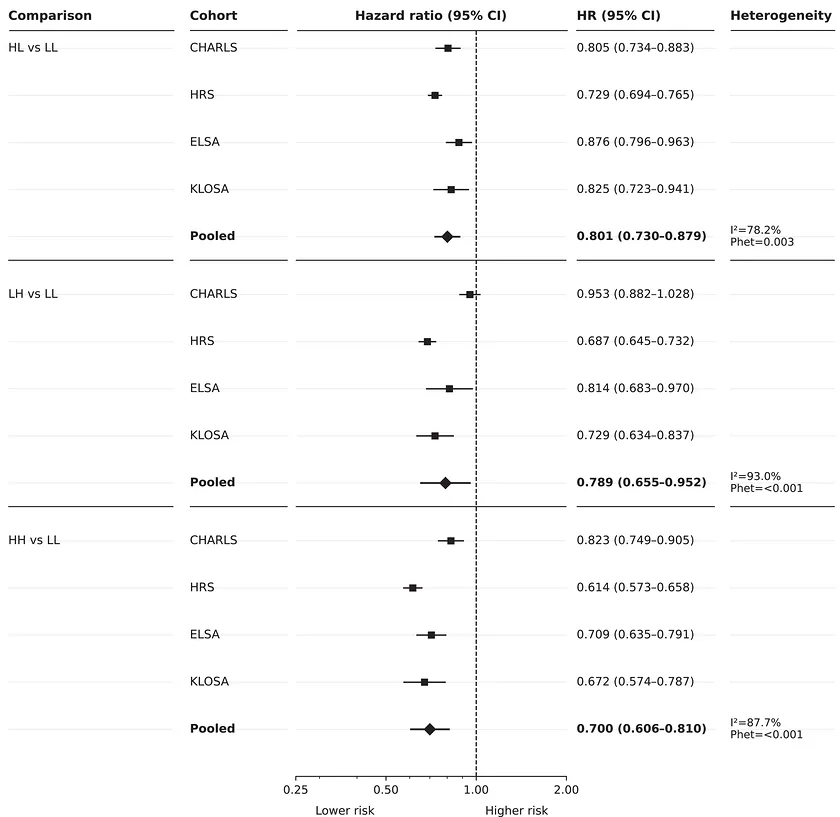
Associations of dual‐exposure groups with incident cognitive impairment. Forest plots show cohort‐specific and pooled hazard ratios and 95% confidence intervals for the associations of time‐updated dual‐exposure groups with incident cognitive impairment. The reference group was low exercise + low cognitive–social engagement. Pooled estimates were derived using random‐effects meta‐analysis. Between‐cohort heterogeneity was quantified using the *I* ^2^ statistic and *P* value for heterogeneity. Values < 1.0 indicate lower risk of incident cognitive impairment compared to the reference group. CHARLS, China Health and Retirement Longitudinal Study; CI, confidence interval; CSE, cognitive–social engagement; ELSA, English Longitudinal Study of Ageing; HH, high exercise + high cognitive–social engagement; HL, high exercise + low cognitive–social engagement; HR, hazard ratio; HRS, Health and Retirement Study; *I* ^2^, inconsistency index; KLoSA, Korean Longitudinal Study of Ageing; LH, low exercise + high cognitive–social engagement; LL, low exercise + low cognitive–social engagement; Phet, *P* value for heterogeneity

Direct pairwise comparisons are shown in Figure 3. High exercise + low cognitive–social engagement did not clearly differ from low exercise + high cognitive–social engagement (HR, 1.01; 95% CI, 0.89–1.16). However, the high–high profile was associated with lower risk than either single‐high profile: HR 0.87 (95% CI, 0.79–0.96) compared to high exercise + low engagement, and HR 0.89 (95% CI, 0.84–0.93) compared to low exercise + high engagement. These findings suggest that structured exercise and cognitive–social engagement were not fully interchangeable, and that maintaining both domains was associated with the most favorable cognitive profile.

**FIGURE 3:**
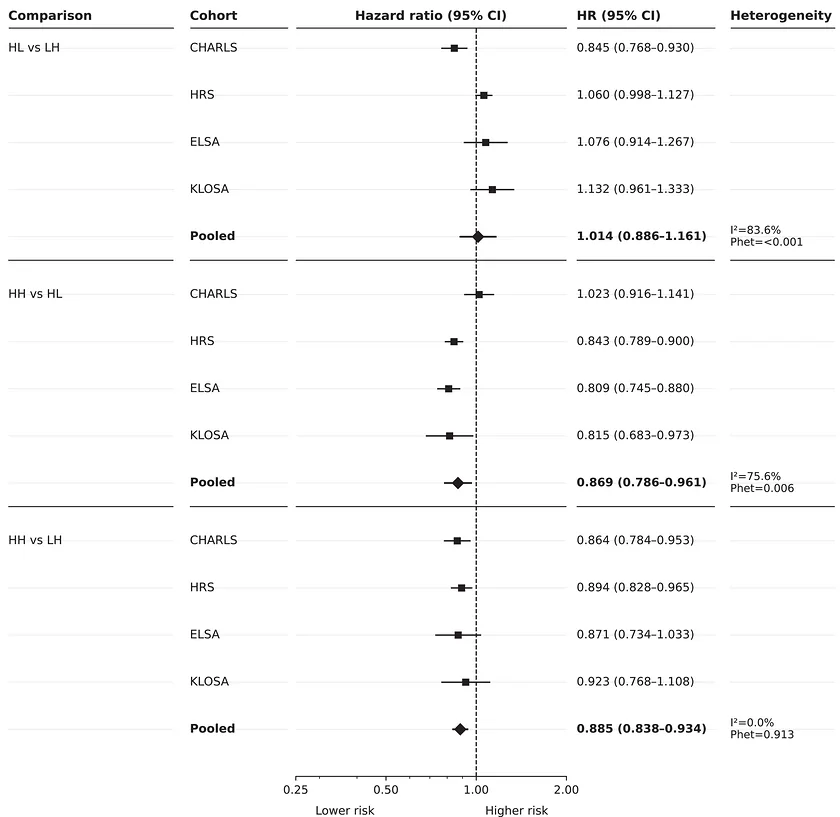
Pairwise comparisons of dual‐exposure groups for incident cognitive impairment. Forest plots show cohort‐specific and pooled hazard ratios and 95% confidence intervals for direct pairwise comparisons between favorable dual‐exposure groups. Comparisons include high exercise + low cognitive–social engagement versus low exercise + high cognitive–social engagement, high exercise + high cognitive–social engagement versus high exercise + low cognitive–social engagement, and high exercise + high cognitive–social engagement versus low exercise + high cognitive–social engagement. Pooled estimates were derived using random‐effects meta‐analysis. Values < 1.0 indicate lower risk in the first group of each comparison. CHARLS, China Health and Retirement Longitudinal Study; CI, confidence interval; CSE, cognitive–social engagement; ELSA, English Longitudinal Study of Ageing; HH, high exercise + high cognitive–social engagement; HL, high exercise + low cognitive–social engagement; HR, hazard ratio; HRS, Health and Retirement Study; *I* ^2^, inconsistency index; KLoSA, Korean Longitudinal Study of Ageing; LH, low exercise + high cognitive–social engagement; LL, low exercise + low cognitive–social engagement; Phet, *P* value for heterogeneity.

### 3.3 Long‐term exercise–engagement patterns

Longitudinal transitions across five waves are shown in Figure 4A. Among 29,758 participants with complete five‐wave exposure data, dual‐exposure profiles changed substantially over time. The low–low group decreased from 10,509 participants at wave 1 to 6098 at wave 5, whereas the high exercise + low engagement group increased from 7630 to 12,845 and the high–high group increased from 5263 to 6110.

**FIGURE 4:**
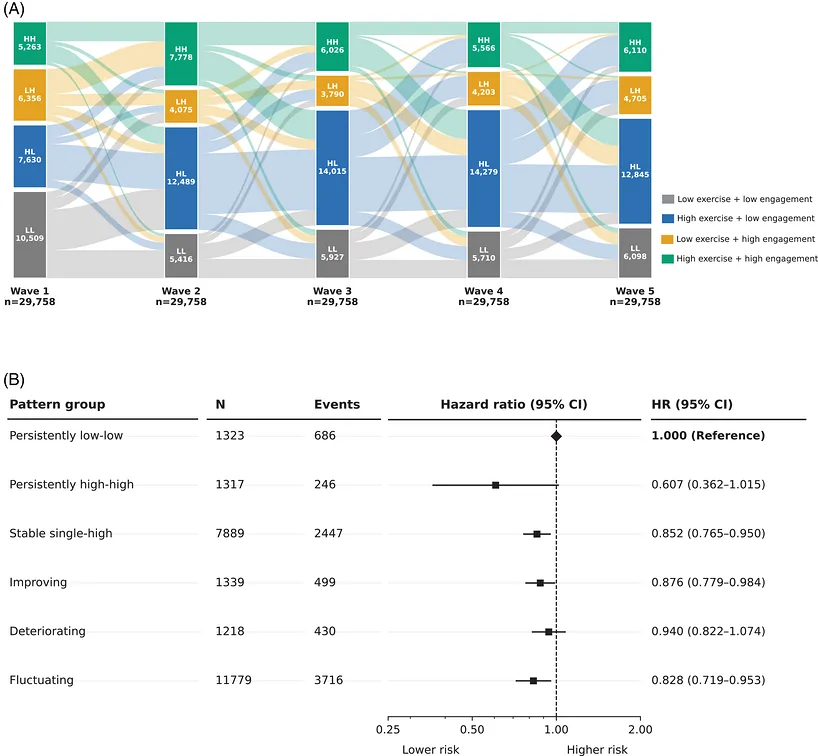
Longitudinal transitions in exercise–engagement profiles and their associations with incident cognitive impairment. A, Alluvial plot showing transitions in the four dual‐exposure categories across the first five observed eligible waves among participants with complete dual‐exposure classification. Flows represent movement between low exercise + low cognitive–social engagement, high exercise + low cognitive–social engagement, low exercise + high cognitive–social engagement, and high exercise + high cognitive–social engagement over time. B, Forest plot showing hazard ratios and 95% confidence intervals for the associations of predominant longitudinal dual‐exposure pattern groups with incident cognitive impairment. The reference group was persistently low–low. Pattern groups included persistently low–low, persistently high–high, stable single‐high, improving, deteriorating, and fluctuating profiles. Values < 1.0 indicate lower risk of incident cognitive impairment compared to the persistently low–low group. CI, confidence interval; CSE, cognitive–social engagement; HH, high exercise + high cognitive–social engagement; HL, high exercise + low cognitive–social engagement; HR, hazard ratio; LH, low exercise + high cognitive–social engagement; LL, low exercise + low cognitive–social engagement.

Predominant five‐wave patterns were associated with subsequent cognitive impairment risk (Figure 4B and Figure S1 in supporting information). Compared to persistently low–low, the lowest point estimate was observed for persistently high–high (HR, 0.61; 95% CI, 0.36–1.02). Stable single‐high, improving, and fluctuating patterns were also associated with lower hazards of cognitive impairment, whereas deteriorating patterns showed weaker evidence of protection. Overall, more sustained favorable profiles appeared to be associated with lower cognitive impairment risk.

### 3.4 Cognitive impairment–free life expectancy

Figure 5 translates these associations into life expectancy with and without cognitive impairment from age 50. Total remaining life expectancy was 28.0 years in the low–low group, 35.7 years in the high exercise + low engagement group, 33.9 years in the low exercise + high engagement group, and 39.1 years in the high–high group.

**FIGURE 5:**
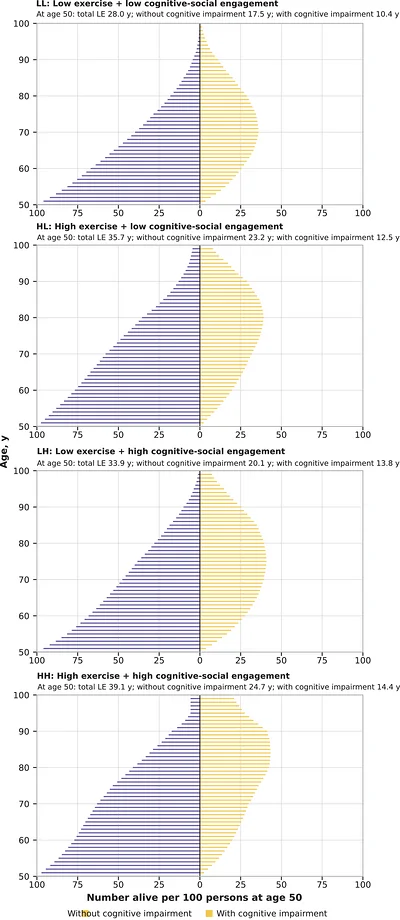
Overall life expectancy and life expectancy with and without cognitive impairment from age 50. Panels show age‐specific state occupancy from a three‐state multistate life table according to dual‐exposure group. The three states were alive without cognitive impairment, alive with cognitive impairment, and death. Widths represent the estimated number alive in each cognitive state at each age per 100 persons at age 50. Text within each panel reports total life expectancy, cognitive impairment‐free life expectancy, and life expectancy with cognitive impairment from age 50. HH, high exercise + high cognitive–social engagement; HL, high exercise + low cognitive–social engagement; LE, life expectancy; LH, low exercise + high cognitive–social engagement; LL, low exercise + low cognitive‐social engagement; y, years.

Cognitive impairment–free life expectancy was 17.5 years in the low–low group, 23.2 years in the high exercise + low engagement group, 20.1 years in the low exercise + high engagement group, and 24.7 years in the high–high group. Thus, compared to low–low, the high–high profile was associated with 7.2 additional cognitive impairment–free years. Compared to low exercise + high engagement, high exercise + low engagement was associated with 3.2 additional cognitive impairment–free years, and the high–high profile was associated with 4.7 additional cognitive impairment–free years. Detailed absolute and proportional estimates are shown in Figures S2–S4 and Table S4 in supporting information.

### 3.5 Sensitivity, subgroup, and supplementary analyses

Sensitivity analyses generally supported the main findings (Figure S5 in supporting information). The HH‐versus‐LL association remained favorable across lagged analyses, alternative exposure cut‐points, and multiple imputation, with HRs ranging from 0.69 to 0.74. The HH‐versus‐LH comparison was also stable, whereas the HL‐versus‐LH comparison remained close to the null. E‐values are shown in Table S5 in supporting information.

Subgroup and leave‐one‐cohort‐out analyses showed broadly consistent directions of association (Figures S6–S7 in supporting information). The high–high profile generally remained favorable across age, education, body mass index, multimorbidity, and sex strata, and results were not driven by any single cohort. In supplementary mortality analyses, all favorable profiles were associated with lower mortality hazards, with the largest reduction observed for the high–high group (Figure S8 in supporting information).

## 4 DISCUSSION

In this harmonized analysis of four longitudinal aging cohorts, structured exercise and cognitive–social engagement were jointly associated with incident cognitive impairment and cognitive impairment‐free life expectancy. Compared to participants with low levels of both domains, those with either high structured exercise or high cognitive–social engagement had lower risk of cognitive impairment, while the high–high profile showed the most favorable overall pattern. Pairwise comparisons further suggested that the two behavioral domains were not fully interchangeable: high cognitive–social engagement alone did not clearly outperform high structured exercise alone, whereas maintaining high levels of both was associated with lower risk than either single‐high profile. In life expectancy analyses, the high–high group had the greatest number of cognitive impairment–free years from age 50. These findings support a dual‐domain prevention framework in which structured physical exercise and cognitive–social engagement provide combined benefits for cognitive aging. Although cognitive engagement and social engagement represent distinct behavioral domains, they were analyzed as a combined cognitive–social engagement construct in this study to enable harmonized comparisons across cohorts with heterogeneous assessment instruments. Our findings are consistent with previous studies linking physical activity to lower risk of cognitive decline and dementia. Cohort studies and meta‐analyses have generally shown that higher physical activity is associated with more favorable cognitive aging, although estimates vary according to activity intensity, domain, timing, and reverse causation control.^5^, ^27^, ^28^ For example, a large Japanese cohort study found that leisure‐time moderate‐to‐vigorous physical activity was associated with lower risk of disabling dementia, while total daily activity showed less straightforward associations, suggesting that the context and purposefulness of activity may matter.^5^ Recent evidence from preclinical AD research also suggests that higher physical activity may be associated with slower cognitive and functional decline among cognitively unimpaired older adults with elevated amyloid burden.^29^ Our study extends this literature by focusing on structured exercise as a distinct domain and by directly comparing it to cognitive–social engagement rather than treating physical activity as a single broad exposure.

Several biological mechanisms may explain why structured exercise was associated with favorable cognitive outcomes. Purposeful exercise is more likely than incidental movement to reach sufficient intensity, duration, and repetition to improve cardiorespiratory fitness, vascular function, insulin sensitivity, blood pressure, and systemic inflammation, all of which are relevant to dementia risk. Exercise may also promote neuroplasticity through neurotrophic pathways, including brain‐derived neurotrophic factor, and may influence hippocampal function, synaptic plasticity, cerebral perfusion, sleep, and mood.^10^, ^30^, ^31^ These mechanisms are biologically plausible but were not directly tested in the present study; therefore, they should be interpreted as potential pathways rather than confirmed mediators. The distinction between structured exercise and broader activity is important because routine daily movement may be beneficial but may not provide the same repeated physiological stimulus as planned exercise.

Our results also align with evidence that cognitively stimulating and socially engaging activities are associated with better cognitive health. The classic study by Verghese et al. showed that leisure activities, particularly cognitively stimulating activities such as reading, board games, and playing musical instruments, were associated with lower dementia risk.^7^ Whitehall II data further suggested that trajectories of cognitive and social activities from midlife to late life were associated with later life cognition and brain health.^14^ Community engagement has also been associated with lower dementia risk in national cohort data, and a meta‐analysis of longitudinal studies found that multiple aspects of social relationships were associated with cognitive decline.^8^, ^32^ These studies support the idea that cognitive–social engagement may help preserve cognitive function, but most examined social, cognitive, or leisure activities separately rather than in direct comparison to structured exercise. In the present study, cognitive and social engagement were considered jointly as a harmonized engagement domain to enable cross‐cohort comparison, while acknowledging that these domains may contribute to cognitive aging through partially distinct pathways.

The potential mechanisms linking cognitive–social engagement to cognitive aging are likely multifactorial. Cognitive activities may enhance cognitive reserve, allowing individuals to maintain function despite age‐related brain changes or neuropathology.^10^ Social engagement may also contribute to “social reserve” through maintaining meaningful ties, emotional support, role identity, and adaptive behaviors.^11^ In addition, social participation may reduce loneliness, depressive symptoms, chronic stress, and hypothalamic–pituitary–adrenal (HPA) axis dysregulation, while also encouraging healthier routines and care‐seeking behaviors.^33^, ^34^ These mechanisms differ from, but may interact with, the vascular and neurotrophic pathways of exercise. This provides a plausible explanation for the combined benefit observed in the high–high group: structured exercise may support brain maintenance and vascular–metabolic health, while cognitive–social engagement may strengthen reserve, stimulation, and psychosocial resilience.

Only a limited number of studies have evaluated physical activity and social engagement together. A recent HRS‐based study among older adults with mild cognitive impairment (MCI) found that both physical activity and social engagement were associated with slower cognitive decline over long‐term follow‐up.^13^ Multidomain intervention evidence also supports the principle that combining exercise, cognitive training, diet, and vascular risk monitoring can improve or maintain cognitive functioning among at‐risk older adults, as shown in the Finnish Geriatric Intervention Study to Prevent Cognitive Impairment and Disability (FINGER) trial.^35^ Our study differs from these works in several ways. First, we focused on incident cognitive impairment among initially unimpaired middle‐aged and older adults, rather than cognitive trajectories among individuals already living with MCI. Second, we used a dual‐exposure framework to directly test whether one favorable domain could compensate for the absence of the other. Third, we translated relative associations into cognitive impairment–free life expectancy, which is more interpretable for clinicians, public health practitioners, and policy makers.

The life expectancy findings provide an important absolute‐risk perspective. Previous work has shown that healthier lifestyle profiles are associated with longer life expectancy and more years lived without AD dementia.^16^ Our study applies a similar framework to the joint role of structured exercise and cognitive–social engagement. The high–high group had the longest cognitive impairment–free life expectancy, and the difference compared to the low–low group was clinically meaningful. However, the high–high group also had longer total life expectancy and more years lived with cognitive impairment, reflecting the fact that longer survival can increase the absolute number of years lived after cognitive impairment onset. Therefore, our findings support an extension of cognitive impairment–free years, but they should not be overinterpreted as definitive compression of cognitive morbidity unless the proportion of remaining life spent free of cognitive impairment is also clearly improved.

Our findings should also be interpreted in the context of between‐cohort heterogeneity. Several pooled estimates showed substantial heterogeneity, particularly for comparisons involving cognitive–social engagement. This is not surprising because engagement activities were harmonized from different survey instruments and may have different cultural meanings across China, the United States, England, and Korea. For example, social participation, internet use, hobbies, religious activities, volunteering, and organization membership may reflect different socioeconomic resources, gender roles, retirement patterns, and community structures in different countries. Nevertheless, the direction of the high–high association was generally favorable, and sensitivity and leave‐one‐cohort‐out analyses did not suggest that the main results were driven by a single cohort. This supports the robustness of the overall interpretation while acknowledging that the magnitude of association may vary across settings.

These findings have implications for dementia prevention. Current public health messages often emphasize physical activity, social participation, or cognitive stimulation as separate lifestyle recommendations. Our results suggest that dementia‐prevention strategies may benefit from a dual‐track approach that promotes structured exercise together with sustained cognitive and social engagement. This is consistent with the broader prevention framework emphasized by the Lancet Commission, which highlights physical inactivity and social isolation as modifiable dementia risk factors.^2^ A practical implication is that community‐based interventions should not only encourage older adults to “be active,” but should also provide structured, purposeful exercise opportunities alongside group‐based, cognitively stimulating, and socially meaningful activities.

Several strengths distinguish this study from previous work. We used repeated data from four aging cohorts, harmonized exposure and outcome definitions across countries, modeled dual‐exposure profiles as time‐updated variables, evaluated direct pairwise comparisons, examined long‐term exposure patterns, and estimated cognitive impairment–free life expectancy. This allowed us to move beyond the simple question of whether activity is beneficial and address a more specific public health question: whether cognitive–social engagement can compensate for insufficient structured exercise. The answer from our data appears to be partial rather than complete. Both single‐high profiles were beneficial compared to low–low, but the high–high profile was most favorable.

Several limitations should be considered. First, structured exercise and cognitive–social engagement were self‐reported and harmonized from cohort‐specific items; residual measurement error and cultural differences are likely. Second, cognitive impairment definitions differed across cohorts and may have introduced outcome misclassification. Third, residual confounding and reverse causation cannot be fully excluded, although lagged, sensitivity, subgroup, and leave‐one‐cohort‐out analyses supported the main findings. Although education and marital/partner status were included as harmonized sociodemographic indicators, socioeconomic factors such as income and wealth could not be consistently harmonized across cohorts, and residual socioeconomic confounding may remain. Fourth, the study did not directly measure mechanisms such as cardiorespiratory fitness, brain structure, amyloid or tau pathology, inflammatory markers, neurotrophic factors, or cognitive reserve. Fifth, the observational design precludes causal inference, and the estimated life expectancy differences should be interpreted as model‐based associations rather than guaranteed benefits of changing behavior.

In conclusion, structured exercise and cognitive–social engagement were jointly associated with incident cognitive impairment and cognitive impairment–free life expectancy across four longitudinal aging cohorts. High levels of both domains were associated with the lowest risk and the greatest number of cognitive impairment–free years. Cognitive–social engagement appeared beneficial but did not fully substitute for structured exercise, supporting a dual‐domain prevention strategy that integrates purposeful physical exercise with sustained cognitive and social engagement.

## CONFLICT OF INTEREST STATEMENT

The authors declare no competing interests. Author disclosures are available in the Supporting Information.

## CONSENT STATEMENT

All participants in the original CHARLS, HRS, ELSA, and KLoSA cohorts provided informed consent according to the procedures approved by the relevant institutional review boards or ethics committees. This study was based on de‐identified secondary data and did not include identifiable personal information; therefore, additional consent for the present analysis was not required.

## Supporting information



Supporting Information

Supporting Information

Supporting Information
