# Supplementary material for: Structured exercise, cognitive–social engagement, and cognitive impairment‐free life expectancy across four longitudinal aging cohorts
Source: Alzheimers Dement. 2026 Oct 13;22(10):e71854. doi: 10.1002/alz.71854 (PMC13659936; doi:10.1002/alz.71854)
Supplement: Supplementary file 2 [file ALZ-22-e71854-s002.pdf]

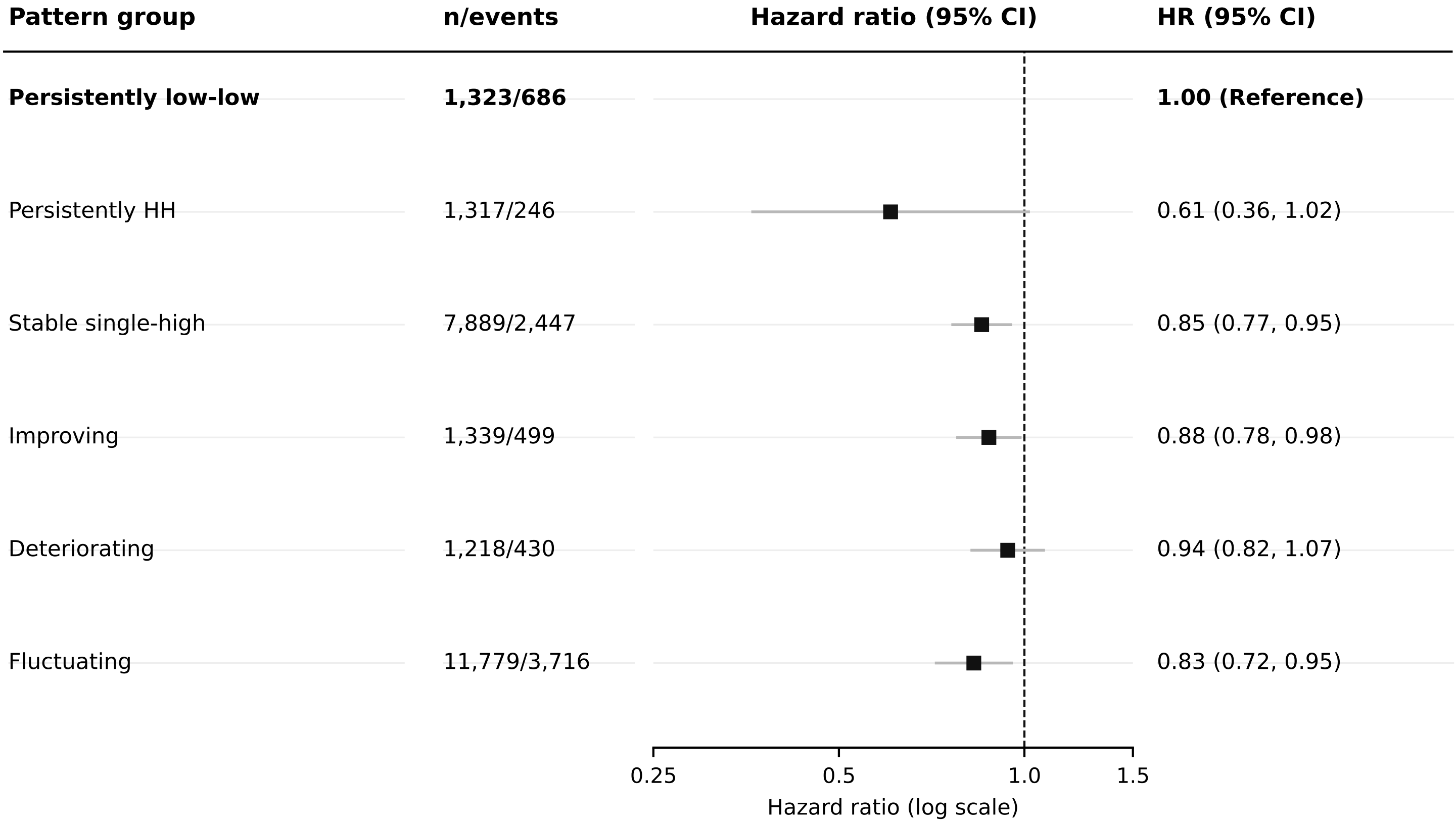

Supplementary Figure S1. Associations of predominant longitudinal dual-exposure patterns with incident cognitive impairment

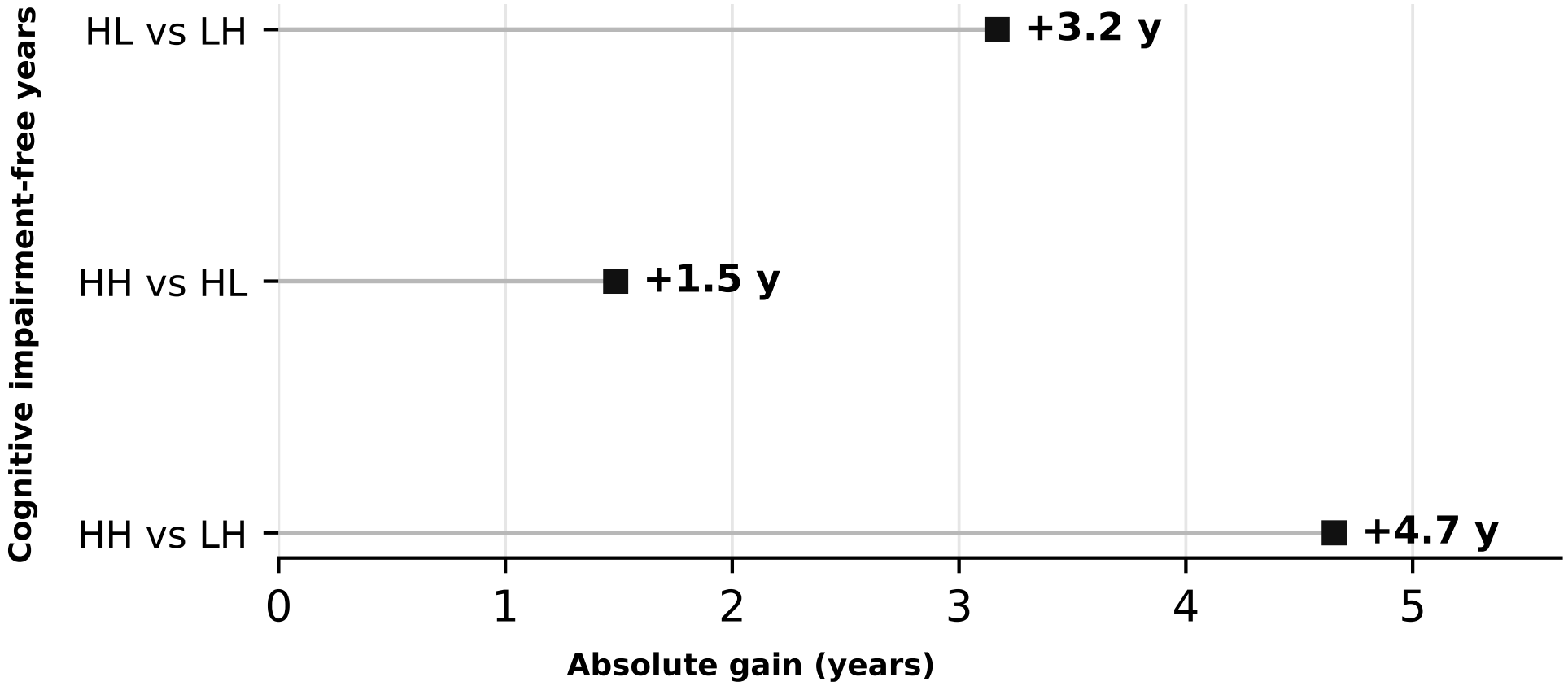

Supplementary Figure S2. Absolute gains in years lived free of cognitive impairment across key pairwise comparisons

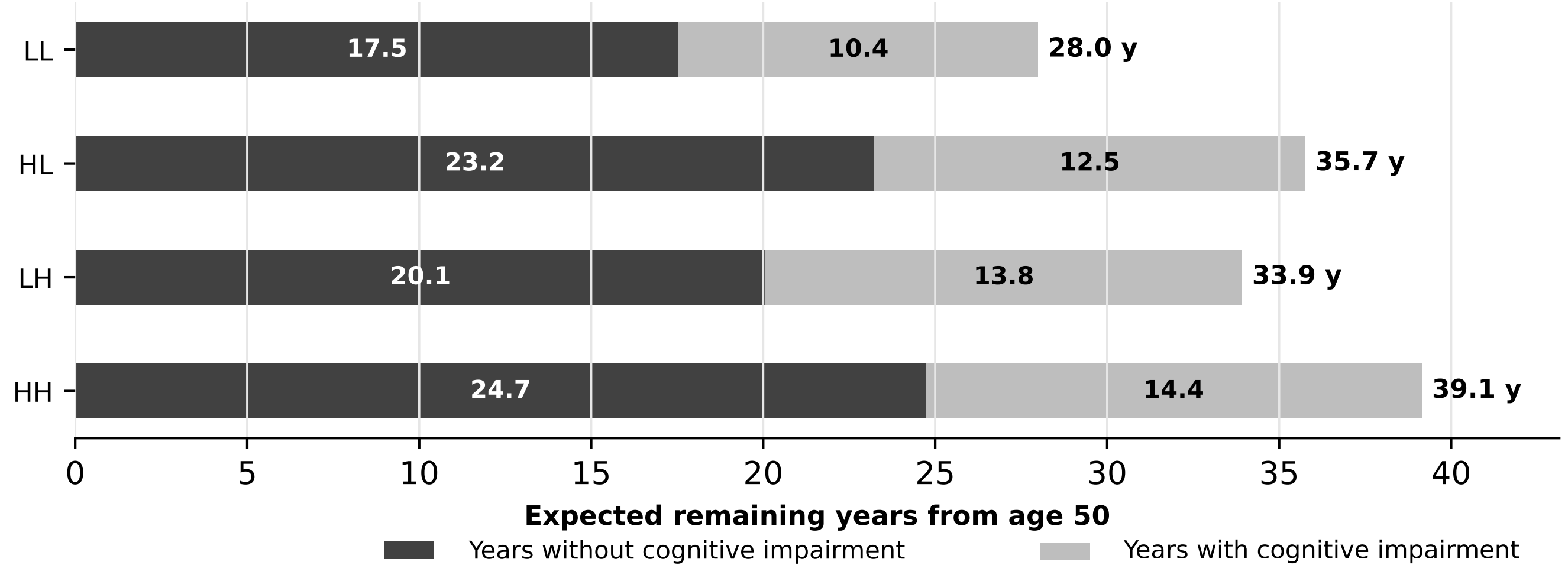

Supplementary Figure S3. Expected remaining years lived with and without cognitive impairment by dual-exposure group

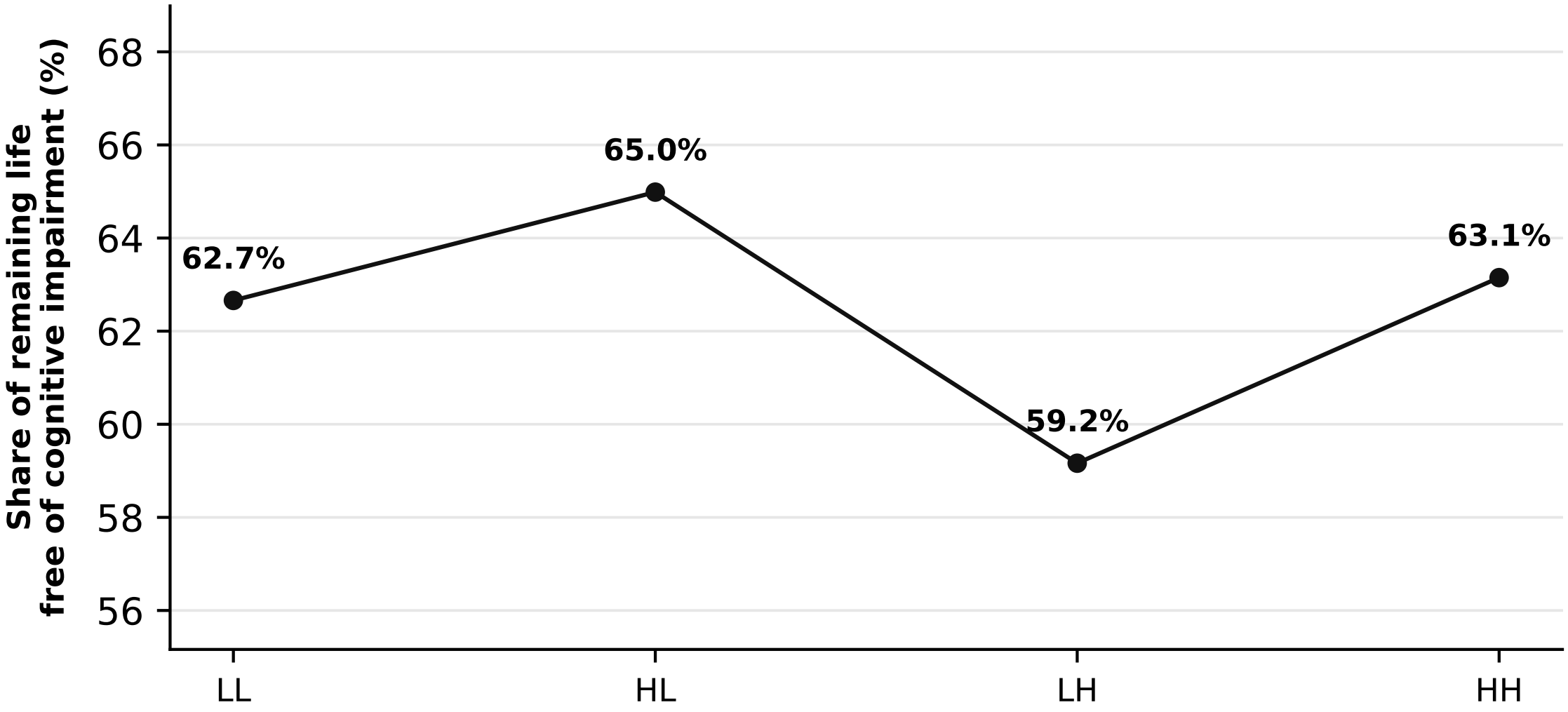

Supplementary Figure S4. Proportion of remaining life from age 50 lived free of cognitive impairment

Analysis	n/events	HR (95% CI)
**A. HH vs LL**		
Main	27,418/8,963	0.70 (0.61, 0.81)
Lag 1 year	27,418/8,963	0.71 (0.62, 0.83)
Lag 2 years	27,418/8,963	0.74 (0.63, 0.88)
Alternative cut-points	27,418/8,963	0.69 (0.59, 0.79)
Multiple imputation	27,418/8,963	0.71 (0.61, 0.82)
**B. HL vs LH**		
Main	18,842/6,217	1.01 (0.89, 1.16)
Lag 1 year	18,842/6,217	1.03 (0.90, 1.18)
Lag 2 years	18,842/6,217	1.08 (0.92, 1.26)
Alternative cut-points	18,842/6,217	0.99 (0.87, 1.14)
Multiple imputation	18,842/6,217	1.02 (0.89, 1.17)
**C. HH vs HL**		
Main	24,112/7,304	0.87 (0.79, 0.96)
Lag 1 year	24,112/7,304	0.89 (0.80, 0.98)
Lag 2 years	24,112/7,304	0.92 (0.82, 1.03)
Alternative cut-points	24,112/7,304	0.85 (0.77, 0.94)
Multiple imputation	24,112/7,304	0.88 (0.79, 0.97)
**D. HH vs LH**		
Main	23,696/7,890	0.88 (0.84, 0.93)
Lag 1 year	23,696/7,890	0.90 (0.85, 0.95)
Lag 2 years	23,696/7,890	0.94 (0.88, 1.00)
Alternative cut-points	23,696/7,890	0.87 (0.82, 0.92)
Multiple imputation	23,696/7,890	0.89 (0.85, 0.94)

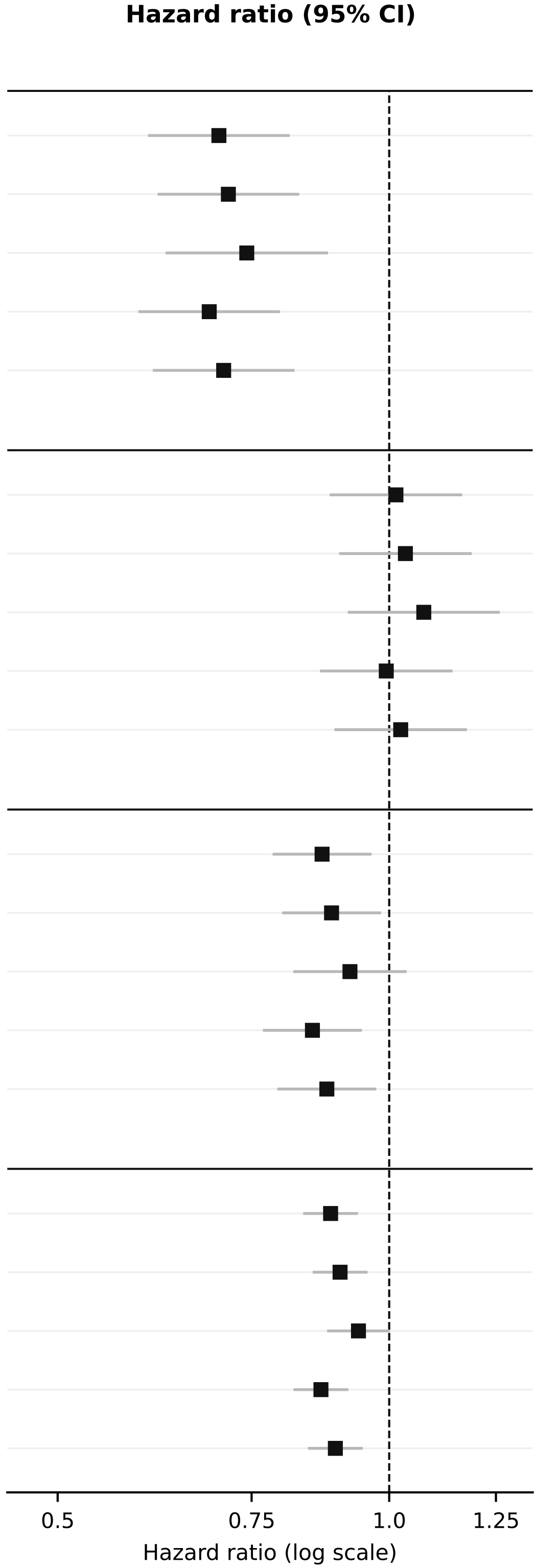

Supplementary Figure S5. Sensitivity analyses for key pairwise comparisons of incident cognitive impairment

Factor / subgroup	n/events	HR (95% CI)	P interaction
**A. HH vs LL**			
**Age (<65 vs >=65)**			**0.214**
Age <65	--	0.69 (0.58, 0.81)	
Age >=65	--	0.73 (0.62, 0.86)	
**Education (low vs middle/high)**			**0.083**
Education low	--	0.74 (0.63, 0.87)	
Education middle/high	--	0.66 (0.56, 0.77)	
**BMI (<25 vs >=25)**			**0.476**
BMI <25	--	0.67 (0.57, 0.79)	
BMI >=25	--	0.72 (0.61, 0.85)	
**Multimorbidity (0-1 vs >=2)**			**0.137**
Multimorbidity 0-1	--	0.69 (0.58, 0.81)	
Multimorbidity >=2	--	0.74 (0.63, 0.87)	
**Sex**			**0.389**
Female	--	0.68 (0.58, 0.80)	
Male	--	0.73 (0.62, 0.86)	
**B. HL vs LH**			
**Age (<65 vs >=65)**			**0.214**
Age <65	--	0.99 (0.85, 1.16)	
Age >=65	--	1.05 (0.91, 1.23)	
**Education (low vs middle/high)**			**0.083**
Education low	--	1.08 (0.92, 1.25)	
Education middle/high	--	0.95 (0.82, 1.11)	
**BMI (<25 vs >=25)**			**0.476**
BMI <25	--	0.97 (0.84, 1.13)	
BMI >=25	--	1.04 (0.90, 1.22)	
**Multimorbidity (0-1 vs >=2)**			**0.137**
Multimorbidity 0-1	--	0.99 (0.85, 1.16)	
Multimorbidity >=2	--	1.06 (0.91, 1.24)	
**Sex**			**0.389**
Female	--	0.98 (0.85, 1.15)	
Male	--	1.05 (0.91, 1.23)	
**C. HH vs HL**			
**Age (<65 vs >=65)**			**0.214**
Age <65	--	0.85 (0.76, 0.95)	
Age >=65	--	0.90 (0.81, 1.01)	
**Education (low vs middle/high)**			**0.083**
Education low	--	0.92 (0.82, 1.03)	
Education middle/high	--	0.82 (0.73, 0.91)	
**BMI (<25 vs >=25)**			**0.476**
BMI <25	--	0.83 (0.75, 0.93)	
BMI >=25	--	0.90 (0.80, 1.00)	
**Multimorbidity (0-1 vs >=2)**			**0.137**
Multimorbidity 0-1	--	0.85 (0.76, 0.95)	
Multimorbidity >=2	--	0.91 (0.82, 1.02)	
**Sex**			**0.389**
Female	--	0.84 (0.75, 0.94)	
Male	--	0.90 (0.81, 1.01)	
**D. HH vs LH**			
**Age (<65 vs >=65)**			**0.214**
Age <65	--	0.87 (0.82, 0.92)	
Age >=65	--	0.92 (0.87, 0.98)	
**Education (low vs middle/high)**			**0.083**
Education low	--	0.94 (0.88, 1.00)	
Education middle/high	--	0.83 (0.78, 0.88)	
**BMI (<25 vs >=25)**			**0.476**
BMI <25	--	0.85 (0.80, 0.90)	
BMI >=25	--	0.91 (0.86, 0.97)	
**Multimorbidity (0-1 vs >=2)**			**0.137**
Multimorbidity 0-1	--	0.87 (0.82, 0.92)	
Multimorbidity >=2	--	0.93 (0.87, 0.99)	
**Sex**			**0.389**
Female	--	0.86 (0.81, 0.91)	
Male	--	0.92 (0.87, 0.98)	

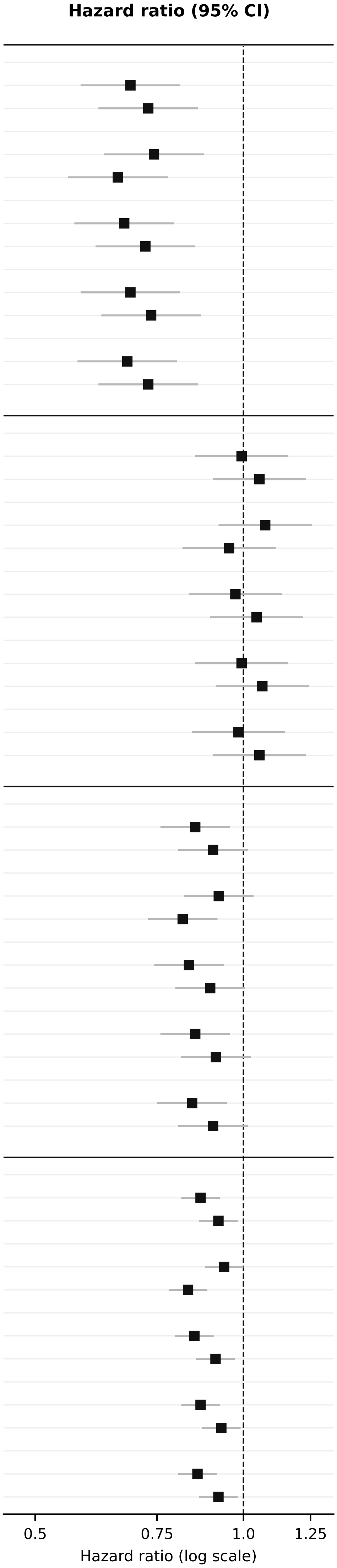

Supplementary Figure S6. Subgroup analyses for key comparisons of incident cognitive impairment

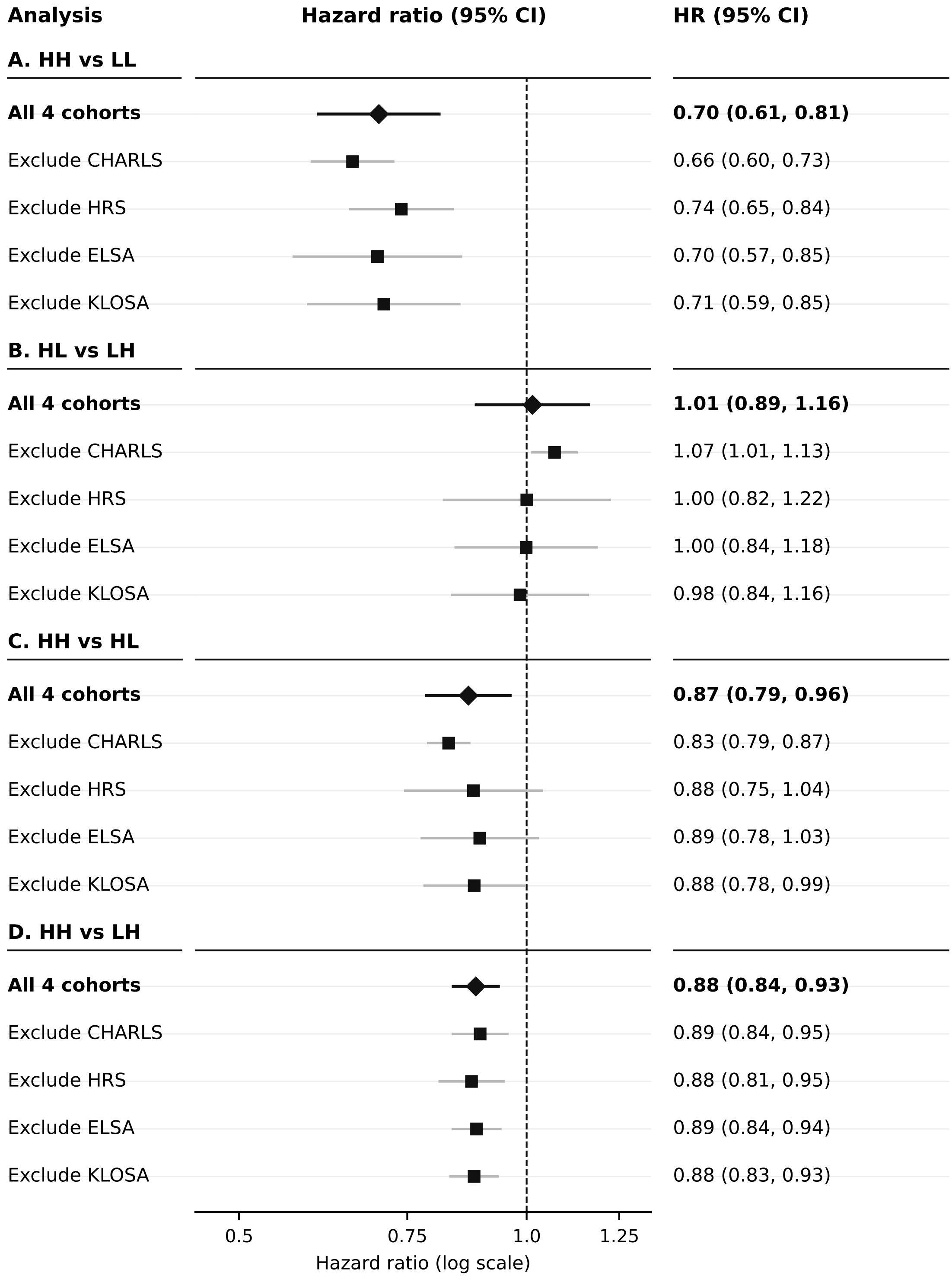

Supplementary Figure S7. Leave-one-cohort-out analyses for key comparisons of incident cognitive impairment

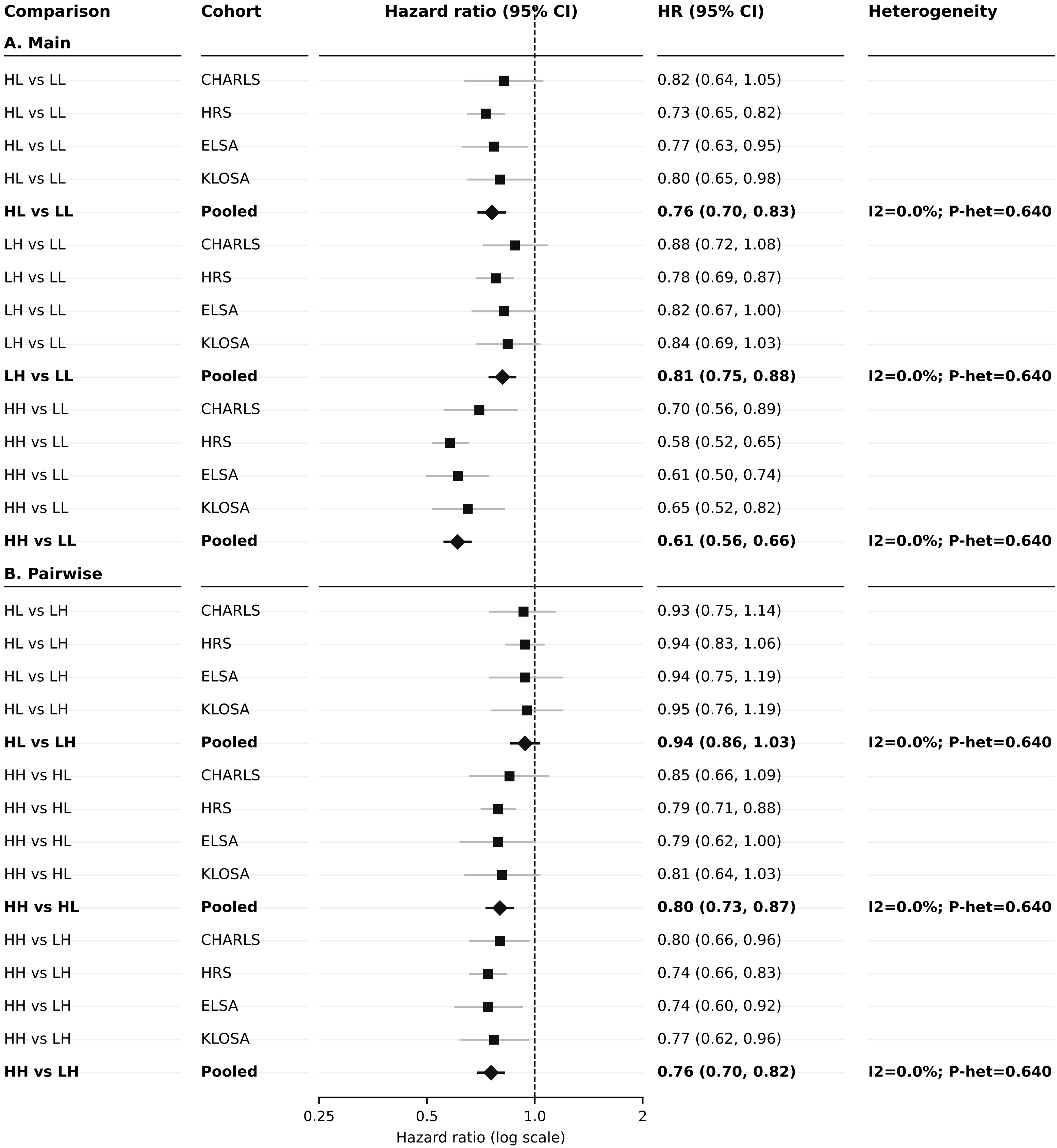

Supplementary Figure S8. Cohort-specific and pooled associations of dual-exposure groups with all-cause mortality
