# Supplementary material for: Structured exercise, cognitive–social engagement, and cognitive impairment‐free life expectancy across four longitudinal aging cohorts
Source: Alzheimers Dement. 2026 Oct 13;22(10):e71854. doi: 10.1002/alz.71854 (PMC13659936; doi:10.1002/alz.71854)
Supplement: Supplementary file 3 [file ALZ-22-e71854-s003.docx]

**Supplementary Table S1. Cohort-specific operationalization of structured exercise and cognitive-social engagement**

| **Cohort** | **Original measure(s)** | **High structured exercise definition** | **Harmonization rationale** | **High cognitive-social engagement definition** | **Harmonization rationale** |
| --- | --- | --- | --- | --- | --- |
| CHARLS | Moderate-to-vigorous physical activity frequency | High if weekly moderate-to-vigorous physical activity was reported. | Categorized according to available frequency information to identify participants with regular purposeful exercise participation. | High if monthly social participation was reported. | Social participation items were harmonized based on available frequency information. |
| HRS | Vigorous physical activity frequency | High if vigorous activity was reported at least monthly/weekly in harmonized frequency categories. | Higher participation categories were used to distinguish relatively more active participants within HRS. | High if monthly or more frequent social activity was reported. | Social activity frequency was categorized using cohort-specific response options. |
| ELSA | Vigorous physical activity frequency | High if vigorous physical activity was reported more than rarely in harmonized frequency categories. | The higher category reflects the upper participation level available in ELSA and does not represent a universal MVPA guideline threshold. | High if organization participation, hobby participation, or weekly internet use was reported. | Multiple engagement domains were combined because available activity measures differed across cohorts. |
| KLoSA | Exercise frequency | High if weekly exercise was reported. | Weekly participation represented the higher available frequency category in KLoSA. | High if weekly social activity was reported. | Social activity frequency was categorized according to available response options. |

Note: Harmonization was conducted based on cohort-specific measures and conceptual similarity of exposure domains rather than identical absolute thresholds across cohorts. Therefore, high and low categories represent relative exposure levels within each cohort and should not be interpreted as universal clinical or guideline-based thresholds.

**Supplementary Table S2.** **Operational definition of wave-specific dual-exposure categories**

| **Category code** | **Structured exercise** | **Cognitive-social engagement** | **Category label** | **Operational interpretation** |
| --- | --- | --- | --- | --- |
| LL | Low | Low | Low exercise + low cognitive-social engagement | Reference profile with low levels of both behavioral domains. |
| HL | High | Low | High exercise + low cognitive-social engagement | High structured exercise but low cognitive-social engagement. |
| LH | Low | High | Low exercise + high cognitive-social engagement | Low structured exercise but high cognitive-social engagement. |
| HH | High | High | High exercise + high cognitive-social engagement | High levels of both structured exercise and cognitive-social engagement. |

Note: LL served as the reference group in the main analyses.

**Supplementary Table S3.** **Operational definition of predominant 5-wave dual-exposure pattern groups**

| **Pattern category** | **Operational definition** | **Conceptual interpretation** |
| --- | --- | --- |
| Persistently low-low | At least 4 of the first 5 eligible waves classified as LL. | Sustained unfavorable profile. |
| Persistently high-high | At least 4 of the first 5 eligible waves classified as HH. | Sustained favorable profile. |
| Stable single-high | At least 4 of the first 5 eligible waves classified as HL or LH. | Stable partially favorable profile. |
| Improving | Not above, with wave 4 and 5 levels higher than wave 1 and 2 levels. | Shift toward a more favorable profile. |
| Deteriorating | Not above, with wave 4 and 5 levels lower than wave 1 and 2 levels. | Shift toward a less favorable profile. |
| Fluctuating | All remaining participants. | Unstable or heterogeneous profile. |

Note: Pattern groups were assigned hierarchically using the first five observed eligible waves.

**Supplementary Table S4. Absolute gains in life expectancy across key pairwise comparisons**

| **Comparison** | **Difference in cognitive impairment-free LE** | **Difference in LE with cognitive impairment** | **Difference in total LE** |
| --- | --- | --- | --- |
| HL vs LH | 3.2 | -1.3 | 1.8 |
| HH vs HL | 1.5 | 1.9 | 3.4 |
| HH vs LH | 4.7 | 0.6 | 5.2 |
| HH vs LL | 7.2 | 4.0 | 11.2 |

Note: Positive values indicate more years in the first group of each comparison.

**Supplementary Table S5. E-values for key pooled comparisons**

| **Outcome** | **Comparison** | **Main HR (95% CI)** | **E-value for point estimate** | **E-value for CI bound** |
| --- | --- | --- | --- | --- |
| Incident cognitive impairment | HH vs LL | 0.700 (0.606, 0.810) | 2.21 | 1.77 |
| Incident cognitive impairment | HL vs LH | 1.014 (0.886, 1.161) | 1.13 | 1.51 |
| Incident cognitive impairment | HH vs HL | 0.869 (0.786, 0.961) | 1.57 | 1.25 |
| Incident cognitive impairment | HH vs LH | 0.885 (0.838, 0.934) | 1.51 | 1.35 |

Note: E-values were calculated for pooled hazard ratios and the confidence-interval bound closest to the null.
